# The curious case of *Prevotella copri*

**DOI:** 10.1080/19490976.2023.2249152

**Published:** 2023-09-01

**Authors:** Nehal Adel Abdelsalam, Shaimaa M. Hegazy, Ramy K. Aziz

**Affiliations:** a Biomedical Sciences, Zewail City of Science and Technology, Giza, Egypt; b Microbiology and Immunology Research Program, Children’s Cancer Hospital Egypt 57357, Cairo, Egypt; c Department of Microbiology and Immunology, Faculty of Pharmacy, Cairo University, Cairo, Egypt; d Center for Genome and Microbiome Research, Cairo University, Cairo, Egypt

**Keywords:** microbiome, diet, metabolism, genomic variation, pharmacomicrobiomics, toxicomicrobiomics, polysaccharide utilization loci, study design

## Abstract

*Prevotella copri* is an abundant member of the human gastrointestinal microbiome, whose relative abundance has curiously been associated with positive and negative impacts on diseases, such as Parkinson’s disease and rheumatoid arthritis. Yet, the verdict is still out on the definitive role of *P. copri* in human health, and on the effect of different diets on its relative abundance in the gut microbiome. The puzzling discrepancies among *P. copri* studies have only recently been attributed to the diversity of its strains, which substantially differ in their encoded metabolic patterns from the commonly used reference strain. However, such strain differences cannot be resolved by common 16S rRNA amplicon profiling methods. Here, we scrutinize *P. copri*, its versatile metabolic potential, and the hypotheses behind the conflicting observations on its association with diet and human health. We also provide suggestions for designing studies and bioinformatics pipelines to better research *P. copri*.

## *Prevotella copri*, a microbiome-derived bacterial species

**Figure 1.**
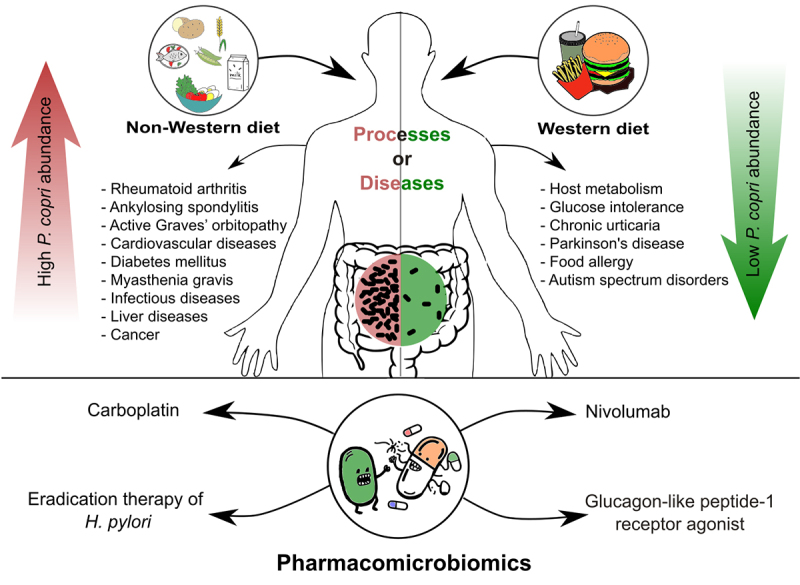
A schematic diagram summarizing the impact of *Prevotella copri* on the human host, with emphasis on the impact of diet, involvement in diseases, and pharmacomicrobiomic interactions. The figure was generated by Inkscape v. 1.3.

Since the emergence of human microbiome studies, almost 20 years ago, the gut and oral microbe, *Prevotella copri*, has been repeatedly detected and mentioned in microbiome profiling studies. Owing to the vast distribution and variable abundance of this intriguing microbe, reports are contradictory about whether it is beneficial or detrimental to human health,^1^ to what extent its impact is on different conditions, and whether these impacts are causal or just associations (Figure 1).

*Prevotella copri* is one of the main species of the *Prevotella* genus and is abundant in the human body, especially in the oral cavity and gastrointestinal tract. It is an anaerobic, Gram-negative, non-spore-forming bacterium.^2^ The name *Prevotella* (derived from the name of the French microbiologist, A. R. Prévot) was officially assigned to the genus in 1990 to distinguish “moderately saccharolytic, predominantly oral *Bacteroides* species” from other *Bacteroides*,^3^ whereas the species *P. copri* (from the ancient Greek *Kopron* = excrement or feces) was only taxonomically recognized in 2007, being among fecal *Prevotella* strains.^4^

Since the earliest human microbiome studies, the two genera *Prevotella* and *Bacteroides* have been observed to be inversely correlated, i.e., high abundance of *P. copri* was often associated with low abundance of *Bacteroides*. This observation led to a popular hypothesis of the presence of different microbiome ‘enterotypes’,^5^ and it was attributed to the fact that both genera belong to the same class and that they strive for the same nutrients.^6–9^

## Nutrition, metabolism, and adaptation to the human gut

*P. copri* can flourish on a range of nutrients, among which are complex polysaccharides. Polysaccharide utilization loci (PULs), proposed by Bjursell et al.,^10^ are sets of genes that encode proteins required for the metabolism and transport of complex polysaccharides in prokaryotes. Their products allow the human gut microbiota to adjust to alterations in host development and host diet.^10,11^ In *P. copri*, such loci are common, and are both distinct and distinctive. In other terms, *P. copri* has unique PULs, which vary among different strains, and their gene products can distinctively break down a wide range of plant-derived (but not animal) polysaccharides in the human gut.^12^

Various studies suggested that *Prevotella* thrives on plant- or fiber-rich diets. These include diets commonly described as non-Western, Mediterranean, or rural African diet.^13,14^ The utilization of plant polysaccharides was compared between *P. copri* grown in culture media alone or with *Candida* spp. While glucose was observed to accelerate *P. copri* growth under no specific conditions, wheat arabinoxylan promoted *P. copri* growth only in a dose-dependent manner and at a slower rate. Starch and carboxymethyl cellulose did not promote *P. copri* growth.^15^

Wheat bran extract, enriched in arabinoxylan oligosaccharides, enhances the growth of *Prevotella* species, especially *P. copri*.^15^ The high abundance of *P. copri* did not correlate with weight loss in participants on an arabinoxylan-oligosaccharide diet.^16^ While wheat bran fermentation was suggested to cause the enrichment of fecal microbiota with *P. copri* in one study,^17^ a diet intervention of wheat peptides and the fucose-rich sulfated polysaccharide fucoidan reduced the relative abundance of *P. copri* and was proposed to alleviate chronic gastritis.^18^ Although dietary fiber might influence *Prevotella*, pinpointing a particular fiber ingredient that can enrich this versatile organism remains challenging.^14^

The Western diet is a high-fat, high-sugar, and low-fiber diet.^19^ It is characterized by a high content of processed food. Limiting advanced glycation end products, which are present in Western diets, significantly reduced the abundance of *P. copri*.^20^ However, intriguingly enough, a study demonstrated that a high relative abundance of *P. copri* was associated with high-protein and Western diet.^21^ The metabolic machinery of *P. copri* might be a great demonstration of its genomic and functional strain-level variability, which allows *P. copri* to easily adapt to the type of nutrients available in the gut: while some strains can degrade carbohydrates and fibers, others can biosynthesize branched-chain amino acids (BCAAs) from meat-based diets.^22^ Recently, a four-week study on rats identified *Prevotella* among the bacteria that slightly decrease with age but are selectively enriched in pomegranate-fed animals.^23^

Another intriguing finding concerning the metabolizing power of *P. copri* is its ability to detoxify superoxide radicals and tolerate reactive oxygen species, which may otherwise increase inflammation.^24^ In a study of the gut microbiome of cyclists, a high abundance of *Prevotella* was associated with amino acid and carbohydrate metabolism.^25^ The inconsistency in *Prevotella*’s response to diet may be due to the interplay between host genetics and microbe-microbe interactions.^17^ Environmental factors and host habits may also contribute to another dimension of variation: geographical variation.^21^

A major player in the gut ecosystem and its microbial composition is bile. Bile acids, the main components of bile – produced by hepatocytes, are important for fat digestion and act as antibacterial agents against most Gram-negative gut bacteria, such as *Prevotella*, *Bacteroides*, and *Parabacteroides*.^26^ Enteric bacteria, by definition, are able to survive bile solubility. Bacteria also modulate the bile production and composition. Deoxycholic acid and lithocholic acid, which are secondary bile acids, are only produced by the action of bacterial enzymes during enterohepatic circulation;^27^ therefore, the gut microbiota composition plays a role in controlling the bile salt pool in the gut.^28^ The role of *P. copri* in the metabolism of bile salts has been previously reported.^29^ The reduction in *P. copri* abundance after the administration of antibiotics in rats resulted in a lower ratio of primary to secondary bile salts.^29^ Consequently, dysbiosis can change bile acid proportions, which are important for the prevention of liver diseases, such as cholestasis, inflammation, fibrosis, and tumors.^30,31^

## In sickness and health: the ups and downs of *P. copri* in the human host

Multiple incongruent conclusions were drawn from research on *P. copri*, which include, but are not limited to, its relative abundance in the human gut, its correlation with diet, its encoded metabolic pathways, and its effect on host metabolism and host health (Table 1). One example is the association between *P. copri* and inflammation and glucose intolerance. Upon metabolizing fibers, *P. copri* produces short-chain fatty acids (SCFAs), which protect the mucosal barrier and reduce the possibility of inflammation.^13^ In one study, the decline in SCFAs was associated with a low abundance of *P. copri*, and therefore suggested to cause inflammatory diseases, such as rheumatoid arthritis, type 1 diabetes (T1D), and T2D.^13^ Yet, other studies have associated a higher abundance of *P. copri* with new-onset rheumatoid arthritis,^43^ or with impaired glucose tolerance.^44^ The latter was explained as an association with the production of BCAAs by *P. copri*.^44^

**Table 1.** Controversial results in studies reporting on *Prevotella copri*. A selection of recent studies on *P. copri*, with emphasis on conflicting results.

Condition	Conclusion	Ref.
Rheumatoid Arthritis (RA) *vs*. Osteoarthritis (OA)	*P. copri* tended to be more abundant in patients with RA, but with no statistical significance.	^32^
	*P. copri* was significantly more abundant in the synovial fluid of patients with RA.	^33^
RA *vs*. Healthy Controls	*P. copri* genes were not detected in healthy controls, but were abundant in patients with early onset RA.	^34^
RA *vs*. Healthy Controls	No difference in relative abundance of *P. copri* between controls and patients on treatment for RA.	^35^
Type 2 Diabetes (T2D)	Patients with T2D suffered from intestinal dysbiosis, marked by an increase of relative *P. copri* abundance	^36^
Type 1 Diabetes (T1D)	*P. copri* was one of the prevalent species in patients with T1D.	^37^
Chronic Urticaria (CU)	At the species level, CU patients had significantly fewer *P. copri* amplicons than healthy controls.	^38^
Parkinson’s Disease (PD)	Relative abundance of *P. copri* decreased in the PD cohort compared with the control group.	^13^
Chronic Liver Disease	*P. copri* was significantly more abundant in patients with advanced fibrosis and suggested to be used as a noninvasive marker for liver fibrosis.	^39^
Human Immunodeficiency Virus (HIV)	In untreated adult patients with HIV-1, *P. copri* was more abundant than in control subjects. *P. copri* was positively associated with CD1c+ mDCs CD40 expression.	^40^
Gastric Cancer	*P. copri* was relatively more abundant in the gastric cancer cohort than the healthy controls. It was suggested as a risk factor for gastric cancer.	^41^
Food Allergy	No food allergies were reported in children whose mothers had *P. copri* in their gut.	^42^

One of the key characteristics of *P. copri* is the expanded heterogeneity between strains.^45^ These divergent strains have been referred to as the “*P. copri* complex”.^46^ Compared with other microorganisms in the human gut, *P. copri* strains vary widely, perhaps because the organism is highly abundant, well-adapted, and widely distributed among humans. The diversity within the *P. copri* complex could be a clue to the *P. copri* paradox in health and disease states,^43^ and consequently, using just one reference strain of *P. copri* limits the exploration of the genomic and functional diversity of other *P. copri* strains.^12^ One study attributed discordance in *P. copri* research to strain differences, microbiome niche, host-microbiome metabolic interactions, and study design.^47^

In the next section, we discuss examples of studies associating relative *P. copri* abundance with human health and disease (Figure 1), highlighting how the conclusions on *P. copri* have been inconsistent and how challenging the research on *P. copri* is.

### Examples in which *P. copri* is less abundant in disease

#### Host metabolism

To investigate the role of the human gut microbiome and diet in regulating human metabolism, Bäckhed et al. studied the effect of barely kernel-based bread (BKB) on glucose metabolism.^8^ In that study, responders were healthy individuals whose insulin and glucose levels improved with BKB consumption, while non-responders were those with least or no improvement in either insulin or glucose. *P. copri* was relatively more abundant in responders in whom complex polysaccharides were metabolized.^8^ When the microbiotas were transferred from responders and non-responders into two groups of germ-free mice, mice with responders’ microbiota had a higher relative abundance of *Prevotella* and enhanced glucose levels. These results suggested that *Prevotella* spp. may be beneficial to human health. Metagenomic analysis of fecal samples from responders and non-responders indicated that responders had a higher relative abundance of microbial genes encoding for complex polysaccharide fermentation. The latter finding was attributed to the capacity of *Prevotella*, specifically *P. copri*, to metabolize complex polysaccharides.^48^

Intestinal gluconeogenesis is a metabolic process that depends on succinate to aid in glucose homeostasis. Therefore, succinate may have metabolic advantages. Although succinate production by *P. copri* was shown to enhance glucose metabolism and insulin levels, succinate levels alone are insufficient to explain the beneficial role of *P. copri* in host glucose tolerance. Therefore, *Prevotella* was suggested to positively affect glucose levels independently of succinate production.^8^ An additional mechanism that may interpret glucose control by *P. copri* is increased bile acid metabolism and farnesoid X receptor signaling. In a study on Goto-Kakizaki rats that underwent vertical sleeve gastrectomy, the authors proposed *P. copri* as a plausible probiotic for T2D because of the abovementioned mechanisms.^29^ In a different study on the microbiome of 1,098 individuals, *P. copri* was found to have a higher relative abundance in individuals with good cardiometabolic markers, such as low visceral fat, high polyunsaturated fatty acids, low C-peptide, and lower postprandial glucose levels.^49^

#### Chronic urticaria

Chronic urticaria (CU) is a skin condition in which patients experience pruritus and sometimes swollen areas that persist for six weeks or longer without a clear cause.^50^ It might be due to the lack of balance in the immune system, which is tightly connected to the gut microbiota. A change in the diversity of the gut microbial community often results in a state of dysbiosis and may trigger inflammatory or allergic reactions.^51–53^ In a study that compared the gut microbiome of healthy individuals with CU, the relative abundance of *P. copri*, among other bacterial species, was significantly lower in patients with CU than in healthy individuals.^38^

#### Parkinson’s disease

Parkinson’s disease (PD) affects the central, autonomic, and enteric nervous systems. Accumulation and aggregation of the neuronal protein, α-synuclein, may contribute to PD propagation.^54^ In addition to a less diverse gut microbial community, the microbiome of patients with PD was found to contain less *P. copri*. Such microbiome imbalance was suggested to stimulate inflammation, Lewy body formation, and α-synuclein aggregation in nerve cells.^55^ Through the vagus nerve, these abnormal proteins can be transferred from the enteric system to the central nervous system.^56^ The past observation was further confirmed in a more elaborate study that used shotgun metagenomics:^13^ patients with early stage PD, who did not receive anti-Parkinson treatment, levodopa (L-DOPA), maintained a significantly lower relative abundance of *P. copri* in comparison with age-matched healthy controls.^13^

#### Autism spectrum disorders

The gut-brain axis is a term that describes the two-way relationship between the gastrointestinal tract and brain in humans. Accruing evidence has established a vital role for the gut microbiota in the communication between the gut and the brain. Consequently, intestinal dysbiosis may be involved in neurological disorders.^57^

A major example of the involvement of microbiota in disease through the gut-brain axis is autism spectrum disorder. According to the National Institute of Mental Health, autism spectrum disorders (ASD) are behavioral and communication disorders in which symptoms may appear in the early years of life.^58^ A comparison of children with ASD with typically cognitive children showed a decreased relative abundance of *P. copri* in the first group.^59^ Although diet is an important element affecting gut microbiota composition, *Prevotella* and *P. copri-*like operational taxonomic units were absent in children with autism suffering from gastrointestinal problems unrelated to their diet.^60^ Intriguingly, in another study, the relative abundance of *P. copri* was significantly higher in children with ASD than that in healthy controls.^61^

#### Food allergy

In the Barwon Infant Study in Australia, the microbiota of mothers and their infants was analyzed, and the infants were tested for different types of food allergies. Remarkably, infants whose mothers carried *P. copri* during pregnancy were at lower risk for food allergy, especially mothers who were on a high-fiber high-fat diet.^42^ Suggested mechanisms behind the observed *P. copri*-associated maternal protection against food allergy development in infants include succinate production, maternal IgG bound to epitopes from *P. copri*, and *P. copri* endotoxin inhibiting Toll-like receptor 4 signaling during the development of the fetal immune system.^42^ Moreover, *P. copri* was reported to be more abundant in healthy controls than in individuals with multiple food allergies, and this observation was explained by SCFA production in healthy controls.^62^

### Examples of *P. copri* enrichment in disease conditions

#### Rheumatoid arthritis

The Centers for Disease Control and Prevention define rheumatoid arthritis (RA) as an inflammatory autoimmune disorder that gradually destructs joint tissues. While the exact reasons behind RA remain unknown, genetic variations between individuals are insufficient to explain RA.^63^ Through distinguishable mechanisms, researchers have advocated the human intestinal microbiota as a vital player in joint diseases, especially in the state of dysbiosis.^32,33,43^

Accruing studies on *P. copri* strongly associate its putative pro-inflammatory characteristics with the development of RA. In a study of patients with new-onset untreated rheumatoid arthritis (NORA), *P. copri* was relatively more abundant in patients with NORA than in healthy controls.^43^ In the same study, a mouse model of gut inflammation demonstrated that *P. copri* augmented chemical colitis, and mice showed more severe disease. The authors suggested that limiting the spread of *P. copri* might halt the incidence or progression.^43^ A recent study demonstrated the prevalence of *P. copri* in the early stages of RA. The authors compared healthy controls with the naïve patients with RA and those receiving disease-modifying antirheumatic drugs (DMARDs).^34^ To explore the association between early RA and *P. copri*, Maeda and coworkers inoculated germ-free SKG mice with fecal microbiota obtained from patients with RA and treated them with zymosan, which triggers the disease.^63^ Severe arthritis in SKG mice was explained by an increase in intestinal T-helper 17 cells. In addition, lymphocytes and *P. copri*-stimulated dendritic cells responded to the arthritis-related autoantigen 60S ribosomal protein L23a by producing interleukin-17.^63^

The interactions between *P. copri* and the immune system, and how these interactions may reflect RA progression have been investigated.^64,65^ During an RA flare of a chronic patient on DMARDs, a peripheral blood mononuclear cell sample contained a *P. copri* HLA-DR-presented peptide. This peptide was predicted to be derived from a *P. copri* 27-kDa protein and is believed to stimulate an immunogenic response via T-helper 1 cells. Both the epitope and the whole *P. copri* organism were able to trigger host antibody responses, which varied between IgA and IgG responses that were only specific to patients with RA rather than other types of arthritis, or to healthy controls. Based on this, one may predict local and systemic immune responses to either *P. copri* or its T-cell epitope. Surprisingly, *P. copri* 16S rRNA genes were detected in the synovial fluid of patients with NORA and chronic RA, albeit at low abundance.^64^ Based on these findings, the authors of that study hypothesized that *P. copri* might continue to circulate from the intestines to the joints, carried by macrophages, during the disease course.^64^ A comparison of the microbiome composition in the synovial fluid of patients with RA with that of patients with osteoarthritis (OA) demonstrated a higher relative abundance of *P. copri* in patients with RA. This finding further supports the unique relationship between the organism and RA.^33^ A recent study reported a correlation between anti-*P. copri* P27 antibodies with autoantibodies found in RA patients and proposed a potential causal role for *P. copri* in RA evolution and synovitis.^66^

Even though the above studies suggested *P. copri* as a key microbe associated with RA, a study on patients receiving treatment for RA found no such association:^35^ Neither the abundance of *P. copri* nor the family *Prevotellaceae* was significantly different between patients with RA and healthy controls.^35^ Methotrexate (MTX) is one of the main therapeutic agents used for the treatment of RA, and its mechanism of action depends on the inhibition of dihydrofolate reductase (DHF). Enzymes involved in tetrahydrofolate biosynthesis were found to be much less expressed in the *P. copri-*enriched microbiome. Therefore, oral MTX had better bioavailability in patients with NORA having *P. copri* in their microbiome, because of the lower competition between the host and microbiota DHF.^43^ Metagenomic analysis of the microbiomes of healthy individuals *vs*. patients with NORA delineated specific open reading frames (ORF) in each cohort. At least two ORFs of the *nuo* operon, coding for components of NADH:ubiquinone oxidoreductase, were associated with healthy status. In contrast, particular ORFs encoding components of an ATP-binding cassette iron transporter were NORA-specific. These distinct ORFs were proposed to serve as microbiome markers to differentiate between healthy and diseased individuals.^43^ Yet, defining an enterotype as a biomarker for RA has remained an unachieved goal, at least until 2019.^67^

The discrepant conclusions of the above studies, if we assume they were performed rigorously, endorse the existence of dissimilar strains of *P. copri* with variable genetic makeup and phenotypes, or dissimilar regulatory programs for *P. copri* strains under different conditions.^68^ Studies started exploring this strain dissimilarity to define signature genes associated with “RA-pathogenic” strains of *P. copri*; those genes belong to the accessory – rather than core – genomes of these strains.^69^

#### Ankylosing spondylitis

Ankylosing spondylitis (AS) is a type of chronic inflammatory arthritis that mainly affects the spine and causes rigidity and fusion of the vertebrae.^70^ In a study on Chinese patients with AS and healthy controls, multiple *Prevotella* species were relatively more abundant in the patient cohort. Among these, *P. copri, P. melaninogenica*, and *Prevotella* sp. C561.^71^ When the fecal microbiota of healthy controls was compared with that of untreated patients with AS and a subset of those patients after treatment, the relative abundance of *P. copri* was found to be higher in patients with AS, but lower after treatment with DMARDs or TNF-alpha inhibitors.^72^

#### Active graves’ orbitopathy

Graves’ orbitopathy (GO) is an autoimmune disorder that threatens the sight of patients with Graves’ disease.^73^ Thyrotropin receptor autoantibody (TRAb) is recommended by the Thyroid Association guidelines as a marker for Graves’ disease and for GO management.^74^ In patients with GO, who had high levels of TRAb, the abundance of the family *Prevotellaceae*, and *P. copri* species, in particular, in the gut microbial community was correlated with TRAb levels. However, the exact mechanism by which *P. copri* influences TRAb levels has not been reported.^75^

#### Diabetes mellitus

Despite studies describing the role of *P. copri* in improving glucose homeostasis, contradicting evidence suggests that the same microbe may be associated with insulin resistance.^44,76^ In individuals with insulin resistance, BCAA levels were high, according to metabolomic analysis. These levels were attributed, at least in part, to the overabundance of *P. copri* in the gut microbiome of these patients.^44^ Functional metagenomic analysis reflected the ability of *P. copri* to synthesize BCAAs, lipopolysaccharides, and tryptophan. Mice developed insulin resistance and a high BCAAs level when orally administered *P. copri*.^44^ A different study confirmed the enrichment of *P. copri* in patients with T2D and suggested *P. copri* as a potential biomarker for the development of this important human disease.^76^

The outer membrane of *P. copri* contains lipopolysaccharide (LPS). Pooled LPS from the microbiome of healthy individuals, especially from members of the order *Bacteroidales*, was found to play a role in tolerating the host immune system.^77^ The surge of LPS in the blood results in a state of metabolic endotoxemia, which results in an inflammatory reaction and insulin resistance.^77^ A positive correlation between BCAAs and LPS in the serum was concluded.^36^ Patients with T2D, especially those on metformin only (rather than metformin and glibenclamide), had a higher relative abundance of *P. copri* than healthy controls. An oral antidiabetic regimen was suggested to influence *P. copri*, and a functional food was proposed to decrease its abundance.^36^

Because the intestinal walls are more permeable in presence of Gram-negative bacteria, a host inflammatory response is expected with *P. copri*. This may explain the poor glucose management reported in patients with T1D, as *P. copri* was more abundant in diabetic patients than in healthy participants.^37^

Two other studies have reported a higher relative abundance of *P. copri* in adult and pediatric patients with T1D.^78,79^

#### Cancer

The link between *P. copri* and different types of cancer remains inexplicit. *P. copri* may have a protective effect against colorectal cancer, as demonstrated by tumor size shrinkage in rats.^80^ While some studies have reported *P. copri* to be one of the causes of colorectal cancer and a useful diagnostic microbial biomarker for colorectal cancer,^81,82^ *P. copri* was reportedly more abundant in the fecal microbiomes of healthy individuals than those of patients with colorectal cancer.^83,84^ In a study combining wet-lab investigation and meta-analysis of fecal microbiome data of patients with melanoma, *P. copri* was more abundant in patients with stage I and stage II melanoma than in healthy individuals.^85^ While *P. copri* had a lower abundance in gastric tumor and peritumor microenvironments compared with normal tissue,^86^ patients with gastric cancer had a higher relative abundance of *P. copri* than healthy controls.^41^ Moreover, in a study performed specifically to determine the risk of gastric cancer in the Korean population, participants whose gastric microbiome had *P. copri* were found to be at higher risk of developing gastric cancer than those with *P. copri-*free microbiome.^41^ A set of microorganisms in the salivary microbiome, including *P. copri*, were shortlisted as biomarkers for oropharyngeal and hypopharyngeal cancers.^87^

#### Cardiovascular diseases

To delve into the role of the gut microbiota in cardiovascular diseases with comparable risk factors, researchers investigated the gut microbiome profiles of patients with coronary artery disease and valve calcification. *P. copri* is a key microbe, particularly in patients with valve calcification. Additionally, *P. copri* was positively associated with high levels of low-density lipoprotein, which is a risk factor for cardiovascular diseases.^88^ This finding contradicts the positive correlation reported between the abundance of *P. copri* and high-density lipoprotein cholesterol in another study.^89^ In addition, *P. copri* is significantly more abundant in the gut microbiota of patients with acute cerebral infarction than in healthy individuals.^90^

#### Liver diseases

When lipid droplets accumulate in 5% or more of liver cells, patients develop nonalcoholic fatty liver disease (NAFLD). When accompanied by inflammation, NAFLD evolves into a more aggressive disease state called nonalcoholic steatohepatitis (NASH). NASH may lead to liver fibrosis and, eventually liver cirrhosis. *P. copri* positively correlated with more severe liver fibrosis in children.^24^ A reasonable justification for this correlation is the highly significant increase in the expression of the microbiome-derived inflammatory products. For example, intermediate biomolecules involved in LPS biosynthesis and flagellar assembly discriminated between healthy obese children and children with chronic liver disease, particularly NASH. Despite opposing findings on the role of *P. copri* in liver diseases, *P. copri* may represent a noninvasive predictor of liver fibrosis, particularly in the state of microbiome dysbiosis.^24,39^

As stated above, liver health status is strongly associated with bile-microbiome interactions. In primary sclerosing cholangitis, a liver disease coupled with inflammation in the bile ducts, *P. copri* was negatively correlated with the disease, especially in patients with concurrent inflammatory bowel disease.^91^ Enriching the gut microbiota with *P. copri* was shown to decrease cholestasis and liver fibrosis.^92^ Conversely, in stage-four hepatitis C, *P. copri* was more abundant than in healthy volunteers;^93^ this observation was suggested to be associated with inflammation and high Th17 and IL-17 levels.^93^ Likewise, a recent metagenomic study associated *P. copri* with NASH in subjects with obesity and attributed this risk for NASH to lower abundance of butyrate-producing pathways and possible higher intestinal permeability.^94^

#### Myasthenia gravis (MG)

Liu *et al*. reported a correlation between MG development and dysbiosis in pediatric patients. An increase in the relative abundance of *P. copri* and other microbes in patients with myasthenia gravis causes a reduction in SCFA production. This reduction seems to play a crucial role in MG development.^95^

#### Infectious diseases

##### Listeria monocytogenes

Listeriosis is a foodborne illness caused by the Gram-positive bacterium, *Listeria monocytogenes*. It accounts for massive outbreaks and, in some cases, can lead to severe illnesses, such as sepsis and meningitis. The intestinal microbiota protects against *Listeria monocytogenes* colonization of the gut and against its transfer to the bloodstream.^96^ However, *P. copri* might decrease the thickness of the mucosal barriers, thus enhancing their permeability to *Listeria* and exacerbating inflammation,^97^ most likely because *Prevotella* synthesizes sulfatase enzymes that degrade the intestinal mucosa’s mucin lining.^98^ In contrast, the bacteriocin Lmo2776, produced by *Listeria monocytogenes*, has an inhibitory effect on the growth of *P. copri* and may decrease its abundance, avoiding excessive inflammation.^97^

##### Bacteremia

The first case of blood infection with *P. copri* was reported in an elderly patient with heart failure. Gut microbiome analysis showed that *P. copri* was the most abundant microorganism in the gut, and was thus suggested as a biomarker for failure of the intestinal barrier.^2^ As stated above, dysbiosis may increase gut permeability and translocate some members of the gut microbial community to the blood.

##### Traveler’s diarrhea

To investigate the relationship between the gut microbiome and travelers’ diarrhea, a large study team analyzed the gut microbiome of 43 participants before and after their travel to tropical regions. *P. copri* was more abundant in participants who had diarrhea.^99^ In a symptomatic infection by *Entamoeba histolytica*, the abundance of *P. copri* was reported to be higher than that in asymptomatic infection by the same parasite.^100,101^ Yet, explaining the association between *P. copri* and traveler’s diarrhea is quite confusing because there has been no consensus in the literature on whether *P. copri* may cause or protect against it.^100,102,103^ This discrepancy can be attributed to the vast differences between the probable strains of *P. copri.*

##### Human immunodeficiency virus

Chronic activation of the innate immune system, and thus chronic inflammation, may lead to a worse prognosis for human immunodeficiency virus (HIV). A possible rationale for chronic inflammation is microbial translocation from the gut to the circulatory system together with LPS production. High expression of colonic myeloid dendritic cells (CD40 cells) was positively correlated with HIV viral load and *P. copri* abundance in patients with AIDS, compared with healthy individuals. Similarly, *P. copri* prompts the maturation of myeloid dendritic cells to produce inflammatory cytokines *in vitro*.^40^ Not only does *P. copri* have this signature in naïve HIV patients, but it is also more abundant in children with HIV and on antiretroviral therapy.^104^ Even during treatment, antiretroviral therapy did not protect against increased intestinal permeability, as indicated by the positively correlated soluble CD14, a marker for microbial translocation, with the higher abundance of *P. copri* in the treated cohort.^104^

### Pharmacomicrobiomics and *P. copri*

Pharmacomicrobiomics studies mutual interactions between the human microbiome and medications.^105,106^ *Prevotella* is a gut microorganism with several documented pharmacomicrobiomic interactions. Carboplatin is an anticancer agent that may cause inflammation of the intestinal membrane in some patients. It also impacts the composition and diversity of the gut microbiota. In a mouse experiment, the severity of carboplatin-induced mucositis increased with a high abundance of *P. copri*.^107^ Moreover, when mice were pretreated with metronidazole, a drug with antifungal and selective antimicrobial activity against anaerobes, the intensity of the mucosal damage decreased as *P. copri* abundance was reduced. In light of these observations, targeting *P. copri* was suggested as a potential feasible strategy to relieve carboplatin-induced mucositis.^107^

Eradication therapy for *Helicobacter pylori* has been found to increase high-density lipoprotein levels, which are negatively associated with cardiovascular disease risk.^89^ This observation was attributed to disturbance in gut microbiome composition. Intriguingly, the abundance of *P. copri*, unlike several other members of the phylum Bacteroidetes, was positively correlated with high-density lipoprotein cholesterol after eradication therapy.^89^

*P. copri* affects the human response to nivolumab, which is an immunotherapeutic agent (anti-programmed cell death-1) used to treat many types of cancer.^108^ The activity of nivolumab was measured by the number of unique memory CD8+ T cells and natural killer cells after drug use. Patients with non-small cell lung carcinoma receiving nivolumab were classified as responders or non-responders according to the number of immune cells produced. Responders had more diverse gut microbiomes before and during drug treatment than non-responders did. *P. copri* was one of the most abundant microorganisms in the responders’ microbiomes. This was explained by the beneficial role of *P. copri*, which synergizes with nivolumab’s action.^109^

Glucagon-like peptide-1 receptor agonist (GLP-1 RA) is an antidiabetic drug that induces variable responses in individuals. When the gut microbiome composition was compared between responders and non-responders by 16S rRNA amplicon sequencing, *P. copri* had higher relative abundance in non-responders, which has been taken as evidence for a negative correlation between *P. copri* abundance and glycemic control with GLP-1 RA.^110^

## The laborious *P. copri*

This section discusses why studying and reaching conclusions on *P. copri* is toilsome work. This includes both the study design and data analysis.

### Study design

#### Sampling methods

Fecal samples are the most common type of samples used in gut microbiome research. Fecal sampling is noninvasive, inexpensive, and relatively easy. However, fecal samples do not reflect inherent alterations in the microbiota along the gastrointestinal tract and may be more susceptible to environmental contamination than other tissues. Other sampling techniques, such as mucosal biopsy, intestinal fluid collection, and rectal swabs, have been reviewed elsewhere.^111^

Suitable storage conditions for fecal samples play a major role in obtaining reliable research results. These conditions, if not well maintained and kept consistent, may introduce variability across studies. The anaerobic nature of *P. copri* adds another dimension to the complexity of its sample collection and storage. The cultivation of noncommercial *P. copri* strains is prohibitive because of their intolerance to oxygen and slow growth.

#### Confounding factors

Most microbiome studies focused on the association between the human microbiome and disease state. Researchers aspire to find straightforward interrelations between microbes and illnesses, so that alternative therapies can be introduced to patients. Nevertheless, numerous confounding factors can stand against this goal. For instance, host-microbiome and microbiome-microbiome interactions are copious, and fluctuate between individuals and within the same individual. Geographical location has striking consequences on an individual’s lifestyle, eating habits, and even microbiome evolution. Although researchers invest considerable efforts to design controlled microbiome studies and minimize confounding variables, recruiting healthy individuals or patients with similar microbiota for microbiome studies can be challenging. Other than strain variations, an additional explanation for the vastly contradictory results between the above studies is the variability in confounding factors; thus, we have indicated experimental approaches and analysis strategies in selected studies (Table 2).

**Table 2.** Different statistical and bioinformatics approaches used in studying *P. copri.*

Condition	Approach	Analysis	Observation	Ref.
NORA, Chronic RA	16S rRNA	GraphPad Prism 6	↑ abundance in synovial fluid	^64^
RA *vs*. healthy	V3-V5 16S rRNA regions	IM TORNADO/LEfSe	No difference in abundance	^35^
AS *vs*. RA and Behcet’s Disease	Metagenomic shotgun sequencing	In-house pipeline	↑ abundance in AS cohort ↓ abundance in patients with AS receiving TNFi or DMARDs rather than NSAIDs only	^72^
T1D	V3-V4 16S rRNA regions	QIIME	↑ prevalence in patients with T1D	^37^
T2D	Whole genome sequencing	R packages	*P. copri* enhanced glucose tolerance	^29^
PD	V3-V4 16S rRNA region	QIIME/ANOSIM/STAMP software	↑ abundance in control cohort	^55^
Active Graves’ Orbitopathy	V4 16S rRNA region	Co-occurrence Network Analysis. Weighted Gene Coexpression Network Analysis	↑ *P. copri* with ↑levels of thyrotropin receptor antibody.	^75^
HIV	V3-V4 16S rRNA regions	QIIME/LEfSE	↑ *P. copri* in children with perinatally transmitted HIV and on ART	^104^
Bacteremia	V3, V4, V6 16S rRNA regions	MicrobAT system/BLAST	↑ *P. copri* in blood and intestine of patient with heart failure.	^2^
Acute Cerebral Infarction	V4 16S rRNA region	QIIME/PLS-DA	↑ *P. copri* in disease cohort	^90^
Myasthenia Gravis	Wholegenome shotgun sequencing	In-house bioinformatics pipeline.	↑ *P. copri* in pediatric patients with MG	^95^

↑ = increase; ↓ = decrease; Ref = reference.

#### Sample size

A major limitation in drawing strong conclusions is the sample size of the study. While a large sample size evidently increases the power of a study and may limit the influence of confounding factors, researchers are sometimes limited by resources, funding, compliance of participants, and the nature of the disease under study.

In Table 3, we compile a representative set of studies on *P. copri*, with careful delineation of their type, sample size, and conclusions about *P. copri* abundance. While the study designs can be more comprehensive, we focused on samples in which *P. copri* was detected.

**Table 3.** Sample type and size in selected studies on involvement of *P. copri* in human diseases.

Condition	Samples	Sample Size*	Abundance in Disease State	Ref
RA *vs*. OA	Synovial tissue Synovial fluid	Total: 183 patients RA [*n* = 125] OA [*n* = 58]	↑	^33^
RA *vs*. healthy	Fecal	Total: 31 early RA [*n* = 17] controls [*n* = 14]	↑	^63^
RA *vs*. healthy	Fecal	Total: 72 Control [*n* = 32] RA [*n* = 40]	No difference	^35^
NORA *vs*. healthy	Fecal	114 stool samples [RA and control]	↑	^43^
T2D	Fecal	Total: 80 Healthy [*n* = 22] T2D [*n* = 58]	↑	^36^
T1D	Fecal	Total: 48 Healthy [*n* = 28] T1D [*n* = 20]	↑	^37^
CU	Fecal	Total: 20 Healthy [*n* = 10] CU [*n* = 10]	↓	^38^
Primary sclerosing cholangitis-inflammatory bowel disease (PSC-IBD)	Fecal	Total: 106 PSC [*n* = 11] PSC-IBD [*n* = 32] UC [*n* = 32] HCT [*n* = 31]	↓	^91^
PD	Fecal	Total: 155 PD [*n* = 89] Controls [*n* = 66]	↓	^55^
NAFLD	Fecal	Total: 124 NAFLD [*n* = 87] Obese [*n* = 37]	↑	^24^
ASD	Fecal	Total: 44 children ASD [*n* = 23] neurotypical [*n* = 21]	↓	^59^
MG	Fecal	Total: 99 pediatrics MG [*n* = 53] Healthy [*n* = 46]	↑	^95^

*In these studies, additional samples were sometimes analyzed; however, the number given only refers to samples in which *P. copri* was detected.

↑ = increase; ↓ = decrease; Ref = reference.

#### Animal models

To validate the observations made on human samples and to establish causality, researchers use animal models in which they inoculate the microbe of interest, mostly after depletion of the normal microbiota of the animal, and often after reconstituting the microbiota with certain organisms. They may test for immune responses, as in *P. copri* studies.

Even though animal models, especially mice, can serve this purpose, the influence of differences in physiology is substantial. In addition, the murine and human microbiomes vary widely.^68^ Yet, the greatest strength of animal models is their ability to establish causality, which is usually practically and ethically not feasible in humans. Once an association with a certain organism is established, this organism can be added to the animal microbiome in different amounts, and at different time points. Such analysis can identify whether the studied phenotype is a consequence of the addition of the organism and whether it is dose dependent.

### In silico analysis

Most microbiome studies apply 16S rRNA amplicon sequencing to characterize the human microbiome community. Although efficient, sequencing variable regions of the 16S rRNA gene might be less specific to discern differences between species and is unable to discern strain differences. Species differentiation has been made possible by full 16S rRNA gene sequencing with long-read sequencing strategies, such as nanopore^112^ or single molecule real-time (SMRT) sequencing.^113^ Still, amplicon sequencing cannot describe the functional potential of a significant microbe, notably the substantial proportion of functions encoded by the non-core genome of a particular species or genus. Researchers often seek to explain why a certain microbe is either positively or negatively correlated with the disease; shotgun metagenomic sequencing provides the data needed for comprehensive functional analysis that may guide the understanding of the association between microbes and diseases.

Shotgun metagenomic sequencing, with sufficient sequencing depth, allows *de novo* assembly to unveil new taxa or new strains that may share a similar core genome but a variable accessory genome. Comparative genomics clarifies strain variations at the single-nucleotide level, genomic rearrangement, and loss or gain of genes, plasmids, or operons. This can hugely shape the microorganism’s metabolizing potential, its interaction with the host, its coping with the host immune system, and its dynamic communication with other microbes in the same niche.^43,45^

Exploring the complexity of *P. copri*’s gene pool is only at its early stages. Genomic and phylogenomic studies are accruing to estimate the actual diversity of this human gut-adapted organism. For example, Nii and coworkers^69^ attempted to address this question with reference to the involvement of *P. copri* in disease (specifically RA),^69^ while Lo Presti and colleagues took a phylogenetic approach to link specific clades to the pathogenicity of bowel diseases.^114^

Because of this immense genomic repertoire, so-called reference strains used in disease-association studies are not always informative because strains vary geographically, and because, while reference strains are historically more convenient or first to study, they are not necessarily a true representative of a species, if such a ‘representative strain’ concept ever exists. This reference strain dilemma powerfully accounts for the many discrepancies in research findings regarding *P. copri*.^14^ As reviewed above, the same biological mechanism carried out by *P. copri* could contribute to two opposing conclusions, while two mutually exclusive metabolic pathways carried by *P. copri* have been proven in different studies.^1,12,22^ In one person, a single strain of a species prevails in the microbiome, either stochastically or according to the host lifestyle, and other host and environmental factors that structure the microbiome composition.^45^ *Prevotella*, in general, and *P. copri*, in particular, happened to be highly abundant^115^ and easily detectable microbiome members (since the emergence of culture-independent techniques); thus, they become ‘usual suspects’ behind several studied phenotypes.

Reviewing multiple publications reveals how variable the sequencing approach, and bioinformatic and statistical analyses are between studies (Table 2). We assume that these might inflict an extra layer of variability on the conclusions of the *P. copri* studies.

## A glimpse into the future

A call for best practices in microbiome analysis is as crucial as the rapid advancement in sequencing technologies, and analysis tools and programs. The best practices reinforce the reliability and reproducibility of microbiome studies. The best microbiome analysis practices can pave the way for well-founded meta-analyses, more research hypotheses, and more solid conclusions. Best laboratory practices combined with established and transparent standard operating procedures for analysis can ensure high-quality research and reduce technical errors.

Updating the available databases with curated reference genome sequences of the different strains of *P. copri* will guarantee a better understanding of its association with disease and health and create less confusion. Developing computational tools and algorithms that enhance strain-level resolution will accelerate and empower correlation studies. Focusing on strain-level analyses, whether through genome sequencing of cultured isolates, single-cell genomics, or metagenome-assembled genome sequencing, will help estimate the actual diversity of the *P. copri* complex and the breadth of its genomic repertoire. Using comparative genomics and phylogenomics will allow a precise definition of the core/pan genome and an estimation of the number of key clades/types of this versatile species, respectively. Machine learning algorithms will develop various classifiers to determine signature gene sets, regulons, and hub genes involved in specific association with, or protection from, different disease conditions.

To grasp the sophistication of the ongoing dynamics between the host and its microbiome and within the microbiome itself, one must implement multi-omics analyses and avoid conclusions based on geographically, spatially, or temporally confined studies. With more geographically representative microbiomes sequenced, and with the complexity and heterogeneity of omics data generated, machine learning can be implemented to incorporate data multidimensionality into context, thus distinguishing real disease associations from diet- or geography-based confounders. With all of the above implemented, perhaps one day, primary care physicians will request a routine microbiome analysis through which they can intervene noninvasively, by removing, adding, or modifying specific organisms to improve the quality of human life.

## Abbreviations

AS: Ankylosing spondylitis; ASD: Autism spectrum disorders; BCAAs: Branched-chain amino acids; BKB: Barley kernel-based bread; CU: Chronic urticaria; DHF: Dihydrofolate reductase; DMARDs: Disease-modifying antirheumatic drugs; GLP-1 RA: Glucagon-like peptide-1 receptor agonist; GO: Graves’ orbitopathy; HIV: Human immunodeficiency virus; L-DOPA: l-3,4-dihydroxyphenylalanine or Levodopa; LPS: Lipopolysaccharides; MG: Myasthenia gravis; MTX: Methotrexate; NAFLD: Nonalcoholic fatty liver disease; NASH: Nonalcoholic steatohepatitis; NORA: New-onset untreated rheumatoid arthritis; OA: Osteoarthritis; PD: Parkinson’s disease; PSC-IBD: Primary sclerosing cholangitis-inflammatory bowel disease; RA: Rheumatoid arthritis; SCFAs: Short-chain fatty Acids; T1D: Type 1 diabetes; T2D: Type 2 diabetes; TRAb: Thyrotropin receptor autoantibody
